# Supplementary material for: Machine Learning-Based Gene Expression Analysis to Identify Prognostic Biomarkers in Upper Tract Urothelial Carcinoma
Source: Cancers (Basel). 2025 Aug 11;17(16):2619. doi: 10.3390/cancers17162619 (PMC12385142; doi:10.3390/cancers17162619)
Supplement: Supplementary file 1 [file cancers-17-02619-s001.zip › cancers-3764341-supplementary.pdf]

cancers

MDPI

SUPPLEMENTARY MATERIAL

Table S1. Differentially expressed genes between progressive and non-progressive UTUC patients

Gene symbol	Log2FoldChange	*p*-adj
ACSS3	-5.53	0.032
ANXA9	6.25	0.003
APOC2	-7.52	0.009
ASB7	-8.07	0.008
ATP6V1B2	-5.68	0.010
CLMP	-10.03	0.001
CPED1	-5.99	0.015
CYP20A1	-23.10	0.000
EEF1AKMT2	-6.38	0.010
ELMOD3	3.38	0.005
EMC9	-4.50	0.016
FBXO5	-3.93	0.001
FKBP7	-6.02	0.045
GDF15	4.55	0.010
GGT6	2.99	0.035
GLRX	-2.11	0.014
HBP1	-3.17	0.012
HLA-A	-10.26	0.035
HLA-A	22.01	0.000
HLA-DOB	-25.38	0.000
HLA-DQA1	-23.85	0.000
HLA-DRA	-21.25	0.000
IL15RA	-4.37	0.017
ITM2A	-22.70	0.000
KDELR3	-7.39	0.034
KRTAP5-3	-22.76	0.000
LEAP2	8.35	0.049
LY6E	-3.42	0.010
MBD4	-6.48	0.000
MELK	-6.75	0.000
MRPL27	-4.09	0.018
MT1F	-6.49	0.018
MT2A	-3.70	0.007
MTO1	-22.29	0.000
ODAM	-9.73	0.038
PCLAF	-2.13	0.047
PCOLCE	-5.34	0.001
PDGFC	-8.25	0.001
PIGH	-4.33	0.041
PLAC8	-3.97	0.016
PLAG1	23.43	0.000
PLAUR	-5.91	0.001
PLK1	-4.61	0.017
PLXDC2	-3.92	0.035
RAP1GDS1	-5.39	0.035

RNA5SP122	-22.33	0.000
RNA5SP283	24.29	0.000
RNA5SP366	-25.54	0.000
RNA5SP452	-23.69	0.000
RNF166	-4.96	0.044
RNF168	-3.68	0.032
RPP40	-7.00	0.004
SAMD11	20.67	0.000
SCN1B	-8.35	0.017
SERPINA3	-7.71	0.001
SIRPA	-5.57	0.010
SLAMF8	-4.41	0.047
SLC27A1	-3.42	0.035
SLC27A3	-6.17	0.013
SMIM5	-7.25	0.008
SPOCD1	5.72	0.000
STK17A	-4.82	0.029
STPG1	-5.73	0.000
SULF1	-4.66	0.021
TAC1	-7.28	0.035
TDRD6	22.23	0.000
THAP10	-22.54	0.000
TMEM175	-4.17	0.017
TOMM6	10.05	0.000
TOR3A	-2.64	0.042
TTC23	-2.73	0.047
UCK2	-6.40	0.044
WNT5B	-10.75	0.000
ZCWPW1	-9.58	0.032
ZFP14	4.36	0.007
ZNF701	-2.30	0.029
